# JOINT GOALS IN OLDER COUPLES: ASSOCIATIONS WITH GOAL PROGRESS AND RELATIONSHIP SATISFACTION

**DOI:** 10.1093/geroni/igz038.2916

**Published:** 2019-11-08

**Authors:** Nadine L Ungar, Victoria I Michalowski, Stella Bähring, Denis Gerstorf, Maureen C Ashe, Kenneth M Madden, Christiane A Hoppmann

**Affiliations:** 1 University of British Columbia, Vancouver, British Columbia, Canada; 2 Humboldt University Berlin, Berlin, Berlin, Germany; 3 University of British Columbia and Center for Hip Health and Mobility, Vancouver, British Columbia, Canada

## Abstract

Goals often involve close others such as spouses, but we know little about how this helps or hinders goal progress and what couple consequences arise. To examine these questions, we investigate associations between joint goals, goal progress, and relationship satisfaction by applying multi-level modeling to data from 119 couples (50% female; Mage=71 years). Participants listed their most important goals and reported if they wanted to achieve these together with their partner (self-rated joint goals). 85% self-reported at least one joint goal. Two raters classified goals as “joint” if both partners mentioned the same goal. Positive illusions–i.e., older adults thinking a goal was joint although it was not reported by the spouse–were frequent. Number of joint goals was related to increased goal progress but only for those with low positive illusions, whereas positive illusions were linked to higher relationship satisfaction. We discuss theoretical and practical implications of our findings.

